# Analysis on the pathogenesis and treatment progress of NRG1 fusion-positive non-small cell lung cancer

**DOI:** 10.3389/fonc.2024.1405380

**Published:** 2024-06-18

**Authors:** Hongyan Li, Lina Xu, Hongshun Cao, Tianyi Wang, Siwen Yang, Yixin Tong, Linlin Wang, Qiang Liu

**Affiliations:** ^1^ Oncology Department of Integrated Traditional Chinese and Western Medicine, Shenyang Chest Hospital & Tenth People’s Hospital, Shenyang, Liaoning, China; ^2^ Department of Thoracic Surgery, Shenyang Chest Hospital & Tenth People’s Hospital, Shenyang, Liaoning, China

**Keywords:** neuregulin-1 (NRG1) fusion, non-small cell lung cancer (NSCLC), treatment progress, pathogenesis, future prospects

## Abstract

Lung cancer persistently leads as the primary cause of morbidity and mortality among malignancies. A notable increase in the prevalence of lung adenocarcinoma has become evident in recent years. Although targeted therapies have shown in treating certain subsets of non-small cell lung cancers (NSCLC), a significant proportion of patients still face suboptimal therapeutic outcomes. Neuregulin-1 (NRG1), a critical member of the *NRG* gene family, initially drew interest due to its distribution within the nascent ventricular endocardium, showcasing an exclusive presence in the endocardium and myocardial microvessels. Recent research has highlighted NRG1’s pivotal role in the genesis and progression across a spectrum of tumors, influencing molecular perturbations across various tumor-associated signaling pathways. This review provides a concise overview of NRG1, including its expression patterns, configuration, and fusion partners. Additionally, we explore the unique features and potential therapeutic strategies for NRG1 fusion-positive occurrences within the context of NSCLC.

## 1 Introduction

Lung cancer remains the leading malignancy in terms of global incidence and mortality. Within this spectrum, non-small cell lung cancer (NSCLC) accounts for approximately 85% of cases (1). Despite advancements in targeted therapies for select NSCLC patients, treatment outcomes remain unsatisfactory for a significant number of individuals.

In recent years, there has been a marked increase in the prevalence of lung adenocarcinoma (2), necessitating an in-depth exploration of novel therapeutic strategies. Neuregulin-1 (NRG1), a member of the NRG gene family (3), has emerged as a key player in tumorigenesis and cancer progression. Initially identified in the nascent ventricular endocardium. NRG1 is selectively expressed in the endocardium and myocardial microvessels. Recent research has revealed its intricate participation in various tumors, yielding molecular modifications across multiple tumor-associated signaling pathways (4, 5). The intricate interplay between NRG1 and tumorigenesis has garnered substantial attention, particularly in the context of NSCLC. Notably, NRG1 gene fusions have been recognized as a critical molecular aberration within a subset of NSCLC cases. Understanding the unique characteristics and mechanisms associated with NRG1 fusion-positive NSCLC is essential for the development of targeted therapeutic strategies.

The primary objective of this review is to provide an exhaustive overview of NRG1, exploring its expression patterns, structural attributes, and fusion counterparts. The focus of this investigation is to illuminate the specific traits and ongoing advancements in managing cases of NRG1 fusion-positive NSCLC.

## 2 The expression of the NRG1 gene

NRG1 plays essential roles in cell signaling, proliferation, differentiation, and survival. In normal tissues, the expression of NRG1 is tightly regulated and occurs in various cell types. NRG1 is especially prominent in developing tissues, where it influences organogenesis and cell differentiation (6). During embryonic development, NRG1 is essential for cardiac development and the formation of the nervous system (7). In cancer, alterations in NRG1 expression can have significant implications for tumor growth and progression (8). Moreover, overexpression of NRG1 has been observed in several cancers, including lung cancer, breast cancer, and pancreatic cancer (9–11). Increased NRG1 expression can activate downstream signaling pathways, such as the ERBB2/ERBB3 pathway, which promotes cancer cell proliferation, migration, and survival (12). Notably, NRG1 expression can be modulated by various factors, including growth factors, hormones, and environmental stimuli (13–16). Dysregulation of NRG1 expression can occur through genetic alterations, epigenetic changes, or altered transcriptional regulation, leading to its involvement in cancer development and progression.

NRG1 gene fusion was initially identified in aggressive mucinous lung adenocarcinoma in 2014 (17, 18). Notably, less than 0.3% of the NRG1 gene encodes a protein, and it gives rise to numerous isoforms (19). Due to this complexity, NRG1 is not easily detectable by most DNA-based next-generation sequencing (NGS) techniques. However, RNA-based assays have proven efficacy in detecting NRG1 fusions (20).

Notably, the incidence of NRG1 fusions is relatively low, with the largest published series using RNA-based sequencing detecting NRG1 fusions in only 41 out of 22,000 tumor specimens, resulting in an incidence of 0.2%. Among these cases, NSCLC was the most common tumor type, accounting for 25 cases with an incidence of 0.3% (25/9592) (21). Although subsequent studies have reported NRG1 fusions at low frequencies in various tumor types, NSCLC still exhibits the highest number of cases, and currently, there are no approved targeted therapies specifically designed for NRG1 fusion-positive lung cancers (22–26). In solid tumors, research has found that the frequency of NRG1 fusion tumors was 0.2% (7/3263). The most common histological type was lung adenocarcinoma (n=5) (27).

NRG1 expression plays a pivotal role in the progression of cancer, especially in the context of KRAS mutations (28). In pancreatic ductal adenocarcinoma (PDAC), cancer-associated fibroblasts (CAFs) secrete NRG1, which activates ERBB2 and ERBB3 receptor tyrosine kinases (29). This supports KRAS-independent tumor growth and confers resistance against KRAS inhibitors (30). Similarly, in lung cancer, the SLC3A2-NRG1 fusion gene, often coexistent with KRAS mutations, is subject to ADAM17-mediated cleavage, leading to the release of NRG1. This shedding enhances the activation of the ERBB2-ERBB3 heterodimer and downstream signaling pathways, enhancing cell proliferation and resistance to EGFR kinase inhibitors (31). These findings highlight the significance of NRG1 signaling in oncogenic processes and suggest that targeting the NRG1 pathway, alone or in combination with MEK1/2 or ADAM17 inhibition, may represent a promising therapeutic strategy for cancers with KRAS mutations and NRG1 alterations.

## 3 Pathogenesis of NRG1 fusion-positive NSCLC

NRG1 fusion-positive NSCLC constitutes a comparatively uncommon molecular subtype within the spectrum of lung malignancies, accounting for a small proportion of cases (32). This fusion occurs when the NRG1 gene melds with another gene, culminating in an aberrant fusion protein. This hybrid protein possesses the capacity to incite specific cellular signaling pathways, thereby contributing to the complex etiopathogenesis of lung cancer (33). Although the precise mechanisms underlying the pathogenesis of NRG1 fusion-positive NSCLC remain obscure, several hypothesized mechanisms have come to light:

1) The fusion of NRG1 triggers the perpetual activation of select growth signaling pathways, notably the ERBB family (ERBB1, ERBB2, ERBB3, ERBB4) (34), alongside downstream cascades such as PI3K-AKT and MAPK-ERK (35). NRG1 can interact with ERBB4 resulting in ERBB2/ERBB4 heterodimers (36). The interaction of NRG1 with ERBB 4 and ERBB 3 activates cellular signaling pathways, promoting cell growth and differentiation. NRG1 binds directly to the ERBB3 receptor, which is primarily a dimerization partner due to its limited kinase activity. This binding prompts ERBB3 to dimerize with another member of the ERBB family, often ERBB 2 (HER2), which possesses strong kinase activity. Unlike ERBB 3, ERBB 4 has intrinsic kinase activity. NRG1 can bind directly to ERBB 4, leading to homodimerization or heterodimerization with other ERBB receptors, including ERBB 2. These pathways stand as linchpins for cell growth, viability, and proliferation. The derangement of these pathways can unleash unchecked cell growth and culminate in tumorigenesis (37).

2) The NRG1 fusion also infects the realm of cell adhesion and migratory dynamics (38). The disruption of conventional cell adhesion could potentially lend impetus to cancer cell invasiveness and their capacity to metastasize to distant corners of the body (39).

3) NRG1 fusion can lead to ERBB2/ERBB3 heterodimerization and activation of downstream signaling pathways, such as PI3K-AKT and MAPK-ERK. The MAPK-ERK signaling pathway induces the expression of vascular endothelial growth factor (40–42). Several studies suggest that blocking the activation of PI3K-AKT and MAPK-ERK signaling pathways can inhibit tumor angiogenesis (43, 44). In addition, inactivation of ERBB3 in cancer cells attenuates tumor growth and angiogenesis (45, 46). NRG1 fusion might thus promote angiogenesis, providing tumors with essential nutrients and oxygen, which in turn supports their growth and survival (47).

4) Research indicates that NRG1 fusion-positive tumors express negative or low levels of programmed death ligand (48). This implies the potential for NRG1 fusion-positive tumors to employ immune evasion strategies, thereby evading immune surveillance. Such evasion may facilitate unchecked tumor progression (49).

5) The existence of NRG1 fusion alterations in NSCLC has significant therapeutic implications. Identifying these fusions through molecular profiling is crucial, as it can render the tumor susceptible to targeted therapeutic therapies. The potential strategy involves the use of tyrosine kinase inhibitors (TKIs) targeting the ERBB receptor family, which has demonstrated efficacy in certain cases of NRG1 fusion-positive NSCLC (50–52).

Besides, the oncogenic mechanism in NRG1 fusion-positive NSCLC involves the formation of chimeric genes resulting from chromosomal translocations or inversions, which lead to the constitutive activation of the ERBB family receptors, predominantly HER3 and HER2 (53). NRG1 fusion-positive tumors often exhibit a low tumor mutation burden and low PD-L1 expression, potentially contributing to resistance against immune checkpoint inhibitors (54). Targeted therapies, including bispecific antibodies like Zenocutuzumab (Zeno; MCLA-128) that block HER2/HER3 signaling, are being developed to exploit the dependency of these tumors on the NRG1-driven signaling pathways. In a Korean patient cohort, NRG1 fusion-positive tumors were identified in 0.27% of 8,148 solid tumor cases, with a prevalence of 0.72% in lung cancer patients. The pathological characteristics of these tumors were predominantly adenocarcinomas (55). Notably, the presence of low or absent PD-L1 expression and a low tumor mutation burden (TMB) in these tumors may influence their response to immunotherapies and other targeted treatments.

## 4 Characteristics of NRG1 gene fusion-positive NSCLC

First, NRG1 gene fusion is more commonly observed in lung adenocarcinoma, with invasive mucinous adenocarcinoma (IMA) and acinar adenocarcinoma being the most prevalent pathological subtypes in cases with NRG1 fusions (56). Second, the common fusion partners for NRG1 gene fusion in NSCLC are CD74 and SLC3A2. However, there are several rare fusion partners, including SDC4, FGFR1, ATP1B1, CADM1, DIP2B, F11R, FLYWCH1, ITGB1, KRAS, MDK, MRPL13, PLCG2, RBPMS, TNC, VAMP2, and VAPB (57). Among these, the NRG1-CD74 fusion is the most prevalent (58). Third, CD74-NRG1 fusion has been associated with cancer stem-like properties in immature progenitor-like cells (59). Cancer stem cells are linked to tumor recurrence, metastasis, chemotherapy resistance, and poor prognosis (60, 61). Fourth, regarding overall survival, patients with NRG1 fusions, especially those with mucinous lung adenocarcinoma, have been shown to have reduced survival times compared to those without NRG1 fusions (62). Fifth, NRG1 fusions and KRAS mutations have been considered to be mutually exclusive in IMA. However, recent research suggests that there may be cases with both NRG1 fusions and KRAS mutations, challenging the absolute exclusivity, Il lung IMA KRAS is the only marker that was observed in co-occurrence with NRG1 fusions (63). Sixth, patients with IMA who test positive for NRG1 fusions are predominantly female and have a history of never smoking (64, 65). Finally, tumors with NRG1 fusions frequently exhibit overexpression of ERBB2, ERBB3, and pERBB3 (66).

## 5 Treatment strategy for NRG1 fusion-positive NSCLC

Currently, there are no approved targeted agents specifically designed for NRG1 fusion-positive NSCLC. However, researchers have been exploring various treatment strategies to address this specific molecular subtype. The dysregulation of the NRG1/ERBB3 axis has been implicated in NSCLC progression and therapy resistance, making NRG1 fusion a potential prognostic marker for targeted therapy (67). Targeting ERBB2 Kinase: Blocking the activation of the upstream components of the NRG1 activation pathway, such as ERBB3 and ERBB2, has been a primary focus of therapeutic exploration. Drugs that effectively target ERBB2 kinase, such as afatinib, have shown promise in NRG1 fusion NSCLC (68). Clinical reports have demonstrated significant and durable responses to afatinib in patients with NRG1 fusion-positive NSCLC (69, 70). In patients with NRG1 gene fusions positive NSCLC, afatinib has demonstrated potential therapeutic efficacy. In a retrospective, multicenter, non-comparative cohort study, 40 NSCLC Patients were included; 29 received afatinib. Among NSCLC patients treated with afatinib, the objective response rate (ORR) was 48.3%, with a median duration of response (DOR) of 6.8 months (71). Afatinib provides clear clinical benefits in patients with NRG1 fusion-positive NSCLC, particularly in earlier lines of therapy. However, the study also noted tolerability issues with afatinib treatment, including adverse drug reactions (ADRs) such as diarrhea, which are consistent with the known ADR profile of afatinib. MTOR Pathway Inhibition: Transcriptomic analysis of lung cancer with NRG1 gene fusion has revealed activation of the MTOR pathway. *In vitro* and *in vivo* models have suggested that blocking the MTOR pathway with drugs like rapamycin may be a potential therapy for NRG1 fusion-positive lung adenocarcinoma (72). Pyrotinib: Pyrotinib is an oral, irreversible, pan-ERBB tyrosine kinase inhibitor targeting ERBB1, ERBB2, and ERBB4. It has shown good antitumor activity in ERBB2-mutant NSCLC patients receiving chemotherapy and has been used in combination therapies to target NRG1 fusions (73, 74). Zeno, a bispecific HER2/HER3 antibody, and seribantumab, a monoclonal anti-HER3 agent, have demonstrated promising activity against NRG1-rearranged solid malignancies, including NSCLC (75–77). Current phase II clinical trials are evaluating their efficacy (NCT02912949, NCT04383210) (78, 79). Global eNRGy1 Registry: The establishment of a global registry for NRG1 fusion-positive lung cancer has provided valuable data on treatment outcomes. The registry showed that current chemotherapy, immunotherapy, and targeted therapies are not highly effective for NRG1 fusion-positive NSCLC, highlighting the need for further exploration and development of new treatment strategies (63). It is essential to continue research and clinical trials to identify effective and targeted therapies for NRG1 fusion-positive NSCLC (**Table 1**).

**Table 1:** Demonstrates the activation of signaling pathways by NRG1 and identifies potential therapeutic inhibitors.

Signaling Pathway	Activation by NRG1	Inhibitors	Potential Therapeutic Targets
PI3K/AKT/FOXO	NRG1 binds to ERBB receptor, activating PI3K, which phosphorylates and activates AKT, leading to FOXO inactivation by phosphorylation and nuclear exclusion.	PI3K Inhibitors (Wortmannin, LY294002), AKT Inhibitors (MK-2206, Perifosine), mTOR Inhibitors (Sirolimus, Everolimus, Temsirolimus)	PI3K, AKT, FOXO, mTOR
JAK/STAT	NRG1 binds to ERBB receptor, leading to phosphorylation of JAK, which then phosphorylates and activates STAT proteins, promoting gene transcription for cell survival and proliferation.	JAK Inhibitors (Ruxolitinib, Tofacitinib), STAT Inhibitors (Stattic)	JAK, STAT
ERK/MAPK	NRG1 binds to ERBB receptor, activating the Ras-Raf-MEK-ERK cascade, leading to ERK phosphorylation and activation, which promotes cell proliferation and differentiation.	MEK Inhibitors (Trametinib, Cobimetinib), ERK Inhibitors (SCH772984)	MEK, ERK
mTOR	NRG1 activates mTOR pathway via PI3K/AKT signaling, promoting protein synthesis, cell growth, and survival.	mTOR Inhibitors (Rapamycin, Everolimus, Temsirolimus)	mTOR

## 6 Future prospects and outlook

The understanding of NRG1-mediated activation of ERBB3 and its role in promoting asymmetric dimerization with ERBB1, ERBB2, and ERBB4 has provided valuable insights into the molecular mechanisms of NRG1 fusion-positive malignancies. Targeting ERBB2-ERBB3 signaling has emerged as a promising therapeutic approach for patients with NRG1 fusion-positive cancers. Seribantumab (MM-121) is a fully human IgG2 mAb that can compete with NRG1 for binding to ERBB3 and antagonize receptor signaling. Researchers have designed new cell lines and patient-derived xenograft models with NRG1 fusion, and the results showed that Seribantumab blocks the activation of 4 ERBB family members and downstream signaling. Seribantumab blocks growth and induces apoptosis in NRG1 fusion models of lung cancer *in vitro* and *in vivo* (80). Moreover, Seribantumab monotherapy was well tolerated and safe at all dose levels (81). Zeno, an IgG1 subtype antibody targeting the extracellular structures of ERBB2 and ERBB3, has shown potential in inhibiting phosphorylation of ERBB3 and downstream oncogenic signaling. Phase I studies have confirmed its safety and tolerability (82). A case study reported by Schram et al. demonstrated that a patient with NRG1 fusion-positive NSCLC achieved significant tumor shrinkage with Zeno treatment (83). This promising outcome suggests that Zeno holds potential as a targeted therapy for NRG1 fusion-positive lung cancer. The eNRGy trial, a global multi-center phase I/II clinical trial for NRG1 fusion-positive cancers, including lung cancer, has been initiated (NCT02912949) (79). The efficacy of Zeno was evaluated, with 78% of patients experiencing a reduction in target lesions. Zeno provides robust and durable efficacy in advanced NRG1+ NSCLC, coupled with a favorable tolerability profile. The results of this trial could provide crucial information regarding the potential of Zeno as a targeted therapy for NRG1 fusion-positive malignancies. Continued research and clinical trials are essential to validate the potential of drugs like Zeno and to explore other targeted approaches to improve outcomes for patients with NRG1 fusion-positive tumors. As our knowledge of the molecular mechanisms underlying NRG1 fusions continues to expand, the hope is that targeted therapies will emerge, providing more effective and tailored treatment options for patients with this specific molecular alteration.

## 7 Conclusion

In summary, the involvement of NRG1 in promoting ERBB3-mediated signaling and the formation of oncogenic heterodimers with ERBB1, ERBB2, and ERBB4 highlights the significance of NRG1 fusions in driving abnormal cell proliferation and tumor progression. Furthermore, the association of NRG1 fusions with adverse clinical outcomes, such as tumor recurrence, metastasis, chemotherapy resistance, and poor prognosis, emphasizes the urgent need for targeted therapeutic approaches. Although there are currently no approved targeted agents specifically designed for NRG1 fusion-positive NSCLC, ongoing research has explored potential treatment strategies. Drugs like afatinib, pyrotinib, and Zenocutuzumab have shown promise in preclinical and clinical settings, offering hope for improved therapeutic options in the future. However, more in-depth studies are essential to fully comprehend the molecular mechanisms underlying NRG1 fusions and their impact on tumor development and treatment response. In conclusion, NRG1 fusions represent a promising therapeutic target for the development of antitumor strategies. By unraveling the complexities of NRG1 fusion-positive NSCLC and conducting further research, we can advance the field of precision medicine and ultimately improve patient outcomes.

## Author contributions

HL: Writing – original draft, Writing – review & editing. LX: Writing – review & editing. HC: Writing – review & editing. TW: Writing – review & editing. SY: Writing – review & editing. YT: Writing – review & editing. LW: Conceptualization, Writing – review & editing. QL: Conceptualization, Writing – review & editing.
